# Development of a CRISPR/Cas9 Genome Editing System in Dikaryotic *Ganoderma lucidum* for Targeting Key CYP450 Gene Involved in Triterpenoid Synthesis

**DOI:** 10.3390/jof12030183

**Published:** 2026-03-04

**Authors:** Beibei Dong, Yi Tan, Gen Zou, Na Feng, Linmeng Tang, Jie Feng, Yawen Zhang, Chuanhong Tang, Jingsong Zhang

**Affiliations:** 1 College of Food Sciences & Technology, Shanghai Ocean University, Shanghai 201306, China; bb2497636668@163.com (B.D.); 18712462852@163.com (Y.Z.); 2 National Engineering Research Center of Edible Fungi, Institute of Edible Fungi, Shanghai Academy of Agricultural Sciences, Shanghai 201403, China; judy_1989317@126.com (Y.T.); zougen@sibs.ac.cn (G.Z.); fengna006@163.com (N.F.); tanglinmeng@saas.sh.cn (L.T.); sytufengjie@163.com (J.F.)

**Keywords:** *Ganoderma lucidum*, gene editing in dikaryotic strain, CRISPR/Cas9, *cyp450* gene, triterpenoid synthesis

## Abstract

Currently, most research on CRISPR (Clustered Regularly Interspaced Short Palindromic Repeats) gene editing in edible fungi focuses on monokaryotic strains. However, the biological mechanisms in a monokaryotic state often do not accurately reflect the actual physiological and metabolic conditions of dikaryotic strains. Therefore, this study used two mating-type-compatible monokaryotic strains, L1 and L2, isolated from Ganoderma lucidum ‘Hunong No.1’ G0119, and employed an RNP (ribonucleoprotein)-based CRISPR/Cas9 system to successfully knock out the cyp512a3 gene in strain L2, resulting in the edited strain L2-KO-cyp512a3. The strain was single-crossed with the previously edited L1 strain L1-KO-cyp512a3 in our laboratory to obtain a dikaryotic editing strain that was homozygous at the cyp512a3 locus, named G0119-KO-cyp512a3. UPLC-MS (Ultra Performance Liquid Chromatography–Mass Spectrometry) analysis showed that compared to the starting strain G0119, the dikaryotic editing strain exhibited varying degrees of reduction in the content of eight types of ganoderic acids, including ganoderic acid Me, ganoderic acid P, ganoderic acid T1, etc., with the reduction ranging from 30.5% to 80.1%. To further validate the function of cyp512a3, we overexpressed this gene in the L1 strain. The results showed that the contents of ganoderic acid Mk, ganoderic acid S, ganoderic acid T, and ganoderic acid R in the mycelium were 0.548 ± 0.020, 1.780 ± 0.028, 2.416 ± 0.148, and 0.281 ± 0.016 mg/g (dry weight), which were 1.5 times, 1.3 times, 1.3 times, and 1.3 times that of G0119, respectively. By integrating the results of gene knockout and overexpression, it can be clearly established that cyp512a3 is a key cytochrome P450 gene regulating the biosynthesis of ganoderic triterpenoids in Ganoderma lucidum. This study not only establishes, for the first time, a homologous recombination-based gene editing system in dikaryotic strains of *Ganoderma lucidum*, but also provides a research paradigm based on a dikaryon-editing tool for investigating key life traits of other edible fungi.

## 1. Introduction

*Ganoderma lucidum*, belonging to the phylum Basidiomycota, order Polyporales, family Ganodermataceae, and genus Ganoderma, is commonly known as the “mushroom of immortality” [1]. As a globally recognized fungus for both food and medicinal use, *G. lucidum* has been included in the American Herbal Pharmacopoeia and Therapeutic Compendium, the European Pharmacopoeia, and the Pharmacopoeia of the People’s Republic of China [2]. Modern research has found that *G. lucidum* is rich in various active components such as triterpenes, polysaccharides, nucleosides, alkaloids, and sterols, demonstrating multiple biological activities, including immune regulation, antioxidant properties, anti-tumor effects, and liver protection [3,4]. Among them, triterpenoids are considered one of the most important bioactive constituents of *Ganoderma lucidum*. Based on the differences in functional groups attached to their side chains, triterpenoids can be categorized into acidic triterpenoids (containing carboxyl groups) and neutral triterpenoids (lacking carboxyl groups) [5]. As an important subtype of ganoderma triterpenoids, acidic triterpenoids (also known as ganoderic acids) are formed from the lanosterol skeleton via a series of postmodification reactions, including hydroxylation, methylation, and acetylation [6]. To date, numerous studies have reported their diverse functions: for example, ganoderic acid Mk induces apoptosis in cervical cancer cells [7]; ganoderic acid S promotes platelet aggregation [8]; ganoderic acid T induces apoptosis in lung cancer cells [9]; ganoderic acid Me inhibits tumor growth and lung metastasis [10,11]; and ganoderic acid O exerts cytotoxic effects on 95D cancer cells [12]. Furthermore, depending on the side chain groups and the degree of oxidation, triterpenoids also include various derivatives such as ganoderiol, ganoderal and ganolactone [13].

The biosynthesis of ganoderic acids begins with the conversion of acetyl-CoA to its precursor, lanosterol, via the mevalonate (MVA) pathway [14]. Subsequently, the carbon skeleton of lanosterol undergoes a series of modification reactions to form a diverse array of triterpenoid compounds. In this process, proteins encoded by the cytochrome P450 (CYP450) superfamily act as key oxidases, responsible for introducing hydroxyl, carboxyl, and carbonyl groups onto the carbon skeleton, thereby participating in the structural diversification of ganoderma triterpenes [15]. Some *cyp450* genes have been confirmed to be involved in this pathway [16,17,18,19,20,21,22,23]. However, hundreds of triterpenoid compounds have been identified in *G. lucidum*, and the biosynthetic pathways of most remain unclear. The corresponding *cyp450* regulatory genes and their functions lack systematic analysis, which has become an important research direction in the field of secondary metabolism regulation of *G. lucidum*.

As a large filamentous fungus, the development of genetic manipulation techniques for ganoderma has been relatively slow. Currently, there are few reports on *cyp450* genes involved in triterpenoid synthesis that have been validated in homologous systems in *G.lucidum*. In contrast, *Saccharomyces cerevisiae* can synthesize lanosterol via its endogenous mevalonate (MVA) pathway, and its endoplasmic reticulum structure and post-translational modification system are suitable for the catalytic reactions of CYP450 enzymes. Therefore, it is widely used as a heterologous expression system to explore *cyp450* genes related to triterpenoid synthesis in ganoderma. Currently, the functions of multiple *cyp450* genes (such as *cyp5150l8*, *cyp512w2*, *cyp512u6*, *cyp505d13*, etc.) have been identified in yeast systems, and these genes are involved in the biosynthesis of various triterpenes, including HLDOA (3-hydroxy lanosta-8,24-dien-26-oic acid), ganoderic acid Y, and ganoderic acid ZXYL [16,17,18,19,20,21,22,23]. However, heterologous systems still possess inherent limitations, fundamentally stemming from their inability to fully mimic the natural metabolic environment within *G. lucidum*. On one hand, the system lacks a rich natural substrate library and a complex regulatory network inherent to ganoderma, with substrate supply largely relying on artificial exogenous selection, which significantly limits product diversity. On the other hand, heterologous hosts cannot replicate the spatiotemporal regulatory mechanisms of ganoderma under different growth stages or stress conditions (such as transcriptional regulation and epigenetic modifications), and their endogenous enzymatic systems may interfere with the catalytic activity of the exogenous CYP450 enzymes, affecting reaction specificity and efficiency. Therefore, heterologous experiments primarily reflect the “potential” functions of genes, making it difficult to accurately restore their true biological functions and roles in the natural host. This may be an important reason why the synthetic pathways of key active triterpenoid compounds, such as ganoderic acid A, have not been successfully elucidated in heterologous systems. Developing modern gene-editing technologies such as Clustered Regularly Interspaced Short Palindromic Repeats/CRISPR-associated protein 9 (CRISPR/Cas9), and adopting the strategy of directly conducting functional research on *cyp450* genes within the homologous system of *G. lucidum* are of great significance for systematically elucidating the biosynthetic mechanism of ganoderic acids. Moreover, nearly all the currently reported gene-editing systems for *G. lucidum* have been developed based on monokaryotic strains. However, the monokaryotic state does not accurately reflect the synthesis of ganoderic triterpenoids during the growth of the *G. lucidum* fruiting body. Therefore, establishing a gene-editing system in dikaryotic strains and conducting functional studies on relevant *cyp450* genes hold irreplaceable and significant value for elucidating the biosynthetic mechanism of ganoderic triterpenoids.

This study integrated CRISPR/Cas9 gene-editing technology with gene overexpression techniques, using the *G. lucidum* variety ‘Hunong No.1’ as the research material, to functionally analyze the *cyp450* gene *cyp512a3* (designated as *0Z_04785*). The results confirm that this gene is involved in the biosynthesis of various ganoderic acids, including Me, P, T1, and R, which are derived from the mycelium of *G. lucidum*. Notably, this gene is the first *cyp450* gene to be systematically studied and functionally confirmed in the endogenous system of *G. lucidum*. The findings are not only of significant value for elucidating the biosynthetic pathways of ganoderic acids but also lay an important foundation for the subsequent development of high-activity *G. lucidum* products through directed molecular breeding and synthetic metabolic regulation.

## 2. Materials and Methods

### 2.1. Strain and Protoplast Preparation

The dikaryotic strain *G. lucidum* ‘Hunong No.1’ G0119, along with two mating-type compatible monokaryotic strains L1 and L2 isolated from it, were all provided by the Institute of Edible Fungi, Shanghai Academy of Agricultural Sciences. After activation on PDA (potato dextrose agar) medium (39 g/L PDA powder, BD, Franklin Lakes, NJ, USA), the strains were inoculated into YMG liquid medium (4 g/L yeast extract, Oxoid, Basingstoke, UK; 10 g/L malt extract, Oxoid, Basingstoke, UK; and 4 g/L glucose, SCR, Shanghai, China) for mycelial culture. The mycelia were collected, and protoplasts were prepared using 2% (*w*/*v*) lywallzyme (Guangdong Institute of Microbiology, Guangzhou, China). The obtained protoplasts were resuspended in STC solution (0.55 mol/L sorbitol, 10 mmol/L CaCl_2_, and 10 mmol/L Tris-HCl, pH 7.5) and adjusted to a concentration of 10^7^ protoplasts per 100 μL. All experimental procedures were conducted following the methods reported by Tan et al. [24].

### 2.2. Preparation of sgRNA-cyp512a3 and Cas9 Protein

The genomic DNA of *G. lucidum* strain L2 was extracted using the HiPure Fungal DNA Mini Kit (Magen, Guangzhou, China). The full-length *cyp512a3* gene was amplified using primers L2-*cyp512a3*-F/R (Table S1). The gene sequence of *cyp512a3* in L2 was determined by Sanger sequencing (Genewiz, Suzhou, China). Based on the determined gene sequence of *cyp512a3*, sgRNA targets were screened and identified using the CRISPOR online tool (http://crispor.tefor.net, accessed on 1 July 2024). Subsequently, an in vitro transcription template for sgRNA was constructed, with its structure comprising a T7 promoter, the targeting sequence, and the sgRNA scaffold. The in vitro transcription reaction was carried out using the HiScribe T7 Quick High Yield RNA Synthesis Kit (NEB, Beverly, MA, USA), and the transcription products were purified using the RNA Clean & Concentrator-25 (Zymo Research, Irvine, CA, USA), ultimately yielding sgRNA-*cyp512a3*. The Cas9 protein with a nuclear localization signal was purchased from Novoprotein, Inc. (Shanghai, China).

### 2.3. In Vitro Cleavage Experiment of the RNP Complex

To determine the guiding activity of sgRNA-*cyp512a3*, an in vitro cleavage assay was conducted. According to the instructions for the Cas9 protein (Novoprotein, Shanghai, China), a 20 μL reaction system was established, comprising 200 ng of PCR-amplified fragment, 200 ng of sgRNA-*cyp512a3*, 1 μL of Cas9 protein, and 2 μL of 10× Reaction Buffer, with the volume made up to the required amount using nuclease-free water. The reaction was incubated at 37 °C for 1 h and then terminated by treatment at 70 °C for 10 min. After the reaction, an appropriate amount of loading buffer was added, followed by agarose gel electrophoresis analysis. The specificity of the cleavage activity of the RNP complex was assessed by observing whether the sizes of the cleavage product bands matched the expected sizes.

### 2.4. Co-Transformation of RNP-Donor DNA Mediated by PEG

Using the genome of strain L2 as the template, overlap PCR assembly was performed with 2× EasyTaq^®^ PCR SuperMix (TransGen Biotech, Beijing, China). The assembled fragment consisted of a 500 bp sequence upstream of the *cyp512a3* target site, an *ura3* (*orotidine 5′-monophosphate decarboxylase* gene) expression cassette (comprising a 1000 bp promoter, a 947 bp *ura3* gene, and a 573 bp terminator), and a 500 bp flanking sequence downstream of the target site. The relevant primers are listed in Table S1. This fragment was then ligated into the linearized pCE-Zero vector using the ClonExpress^®^ Ultra One Step Cloning Kit (Vazyme, Nanjing, China).

Assemble the RNP complex on ice. The reaction system (20 μL) comprises: 6 μg (37.5 pmol) of Cas9 protein, 3.6 μg (112.5 pmol) of sgRNA-*cyp512a3*, 2 μL of 10× Reaction Buffer, with the volume made up to 20 μL using Nuclease-free water. Incubate at 37 °C for 15 min.

Referring to the PEG-mediated transformation method established by Tan et al. [24], mix 100 μL of STC solution (containing approximately 1 × 10^7^ protoplasts), 15 μg of donor DNA plasmid, 20 μL of RNP complex, 50 μL of PTC solution, and Triton X-100 at a final concentration of 0.006%, and then subject the mixture to ice bath for 10 min. Subsequently, slowly add 1 mL of PTC solution, mix well, and incubate at 20 °C for 50 min. Finally, mix the transformation mixture evenly with minimal medium (Glucose 20 g/L, SCR, Shanghai, China; magnesium sulfate heptahydrate 0.5 g/L, SCR, Shanghai, China; potassium dihydrogen phosphate 0.46 g/L, SCR, Shanghai, China; dipotassium hydrogen phosphate 1 g/L, SCR, Shanghai, China; vitamin B1 0.125 mg/L, Sangon, Shanghai, China; mannitol 109.3 g/L, SCR, Shanghai, China; low melting point agarose 10 g/L, Shaoxin Biotech, Shanghai, China;, asparagine 20 g/L, BBI, Shanghai, China), pour it onto plates, and culture at 26 °C.

### 2.5. Screening and Validation of Gene-Edited Transformants

Individual germinated colonies were picked from the minimal medium and transferred to fresh minimal medium for secondary screening, followed by incubation at 26 °C. The transformants that passed the secondary screening were inoculated onto PDA medium, and their genomic DNA was extracted. Using this DNA as a template, a fragment encompassing the complete homologous recombination site was amplified with primers V-KO-cyp512a3-F/R (Table S1). After agarose gel electrophoresis detection of the PCR products, bands of the expected size were recovered from the gel and sequenced to screen for transformants that had successfully complemented the ura3 marker gene.

### 2.6. Acquisition and Identification of a Dikaryon-Editing Strain

The monokaryotic edited strains L2-KO-*cyp512a3* obtained in this study and the pre-existing monokaryotic edited strain L1-KO-*cyp512a3* in the laboratory were respectively spotted onto PDA plates with a spacing of approximately 3 cm between them, allowing the mycelia to grow and fuse with each other. Mycelia were then picked from the area where the mycelia made contact and transferred to fresh PDA medium. Microscopic observation was conducted to check for the formation of clamp connections. Meanwhile, PCR amplification was performed on the single-nucleotide polymorphism (SNP) site of the *ura3* gene using primers *ura3*-SNP-F/R (Table S1). By analyzing whether overlapping peaks appeared in the sequencing chromatogram, it was determined whether a dikaryotic edited strain had been obtained.

### 2.7. Construction of Overexpression Plasmid and PEG-Mediated Transformation

**Figure 1:**
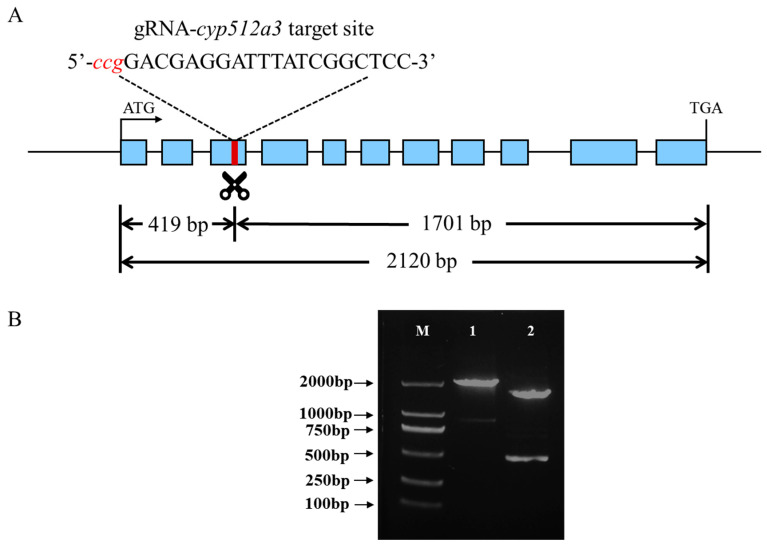
Electrophoretogram of in vitro cleavage of the *cyp512a3* gene by RNP. (**A**) The blue sections represent exons, the white sections represent introns, and the red section represents the target site. (**B**) M indicates the 2000 molecular weight marker; 1 represents the *cyp512a3* gene fragment; 2 represents the two fragments resulting from cleavage by RNP-*cyp512a3*, with the band positions corresponding to 419 bp and 1701 bp, respectively.

To achieve overexpression of the *cyp512a3* gene in strain L1, the expression plasmid pMD-EXP-*cyp512a3* was constructed. Using pMD19T as the backbone, the P*gpd* (promoter of the *glyceraldehyde-3-phosphate dehydrogenase* gene)-intron fragment and the T*trpC* (terminator of the *tryptophan synthase* gene from *Aspergillus nidulans*)-*sdhB* (iron-sulfur protein subunit of the *succinate dehydrogenase* gene) fragment were amplified from the plasmid pMD-EXP-cas9 [25]. Additionally, the *cyp512a3* gene was amplified from strain L1. The expression plasmid was assembled using overlap PCR and the ClonExpress^®^ Ultra One Step Cloning Kit (C115, Vazyme, Nanjing, China) (Figure 1A). The primers used are listed in Table S1. The selection marker of this plasmid is a mutated *sdhB* gene, which confers carboxin resistance to the strain. The plasmid was cloned and propagated in Escherichia coli DH5α. The expression plasmid pMD-EXP-*cyp512a3* (experimental group) and the starting plasmid pMD19T (negative control group) were separately transferred into the protoplasts of strain L1 using a PEG-mediated method: 100 μL of STC containing L1 protoplasts, 10 μg of the pMD-EXP-*cyp512a3* plasmid, and 50 μL of PTC (600 g/L PEG 4000, 10 mmol/L Tris-HCl at pH 7.5, and 50 mmol/L CaCl_2_) were mixed together. After incubation on ice for 10 min, 1 mL of PTC was added and gently mixed, followed by incubation at room temperature for 30 min. Add the transformation system to YMGAM medium (YMG medium supplemented with 10 g/L low-melting-point agarose, Shaoxin Biotech, Shanghai, China; and 109.3 g/L mannitol, SCR, Shanghai, China) for a 48 h recovery period. Then, add the upper screening YMGAM medium containing carboxin (with a final mass concentration of 4 μg/mL) and culture at 26 °C. Subsequently, subculturing was carried out, and re-screening was performed using a PDA medium containing carboxin. The genomic DNA of the resistant transformants was extracted using the HiPure Fungal DNA Mini Kit (Magen, Guangzhou, China). The target band was amplified using primers V-OE-*cyp512a3*-F/R (Table S1) and subjected to sequencing for verification.

### 2.8. Gene Expression by Quantitative Real-Time PCR (qRT-PCR) Analysis of Transformants

The resistant transformants from the experimental group (transformed with the pMD-EXP-*cyp512a3* plasmid), the resistant transformants from the negative control group (transformed with the pMD19T plasmid), and the wild-type strain L1 were respectively inoculated onto PDA medium. After incubation at 26 °C for 10 days, the mycelia were collected. Total RNA was extracted using the HiPure Fungal RNA Mini Kit (Magen, Guangzhou, China). Genomic DNA was removed and reverse-transcribed into cDNA according to the instructions of the FastKing RT Kit (With gDNase) (Tiangen, Beijing, China). Fluorescent quantitative PCR reactions were performed using the SuperReal PreMix Plus (SYBR Green) Kit (Tiangen, Beijing, China). The experiment utilized the *RPL4* gene of *G. lucidum* as an internal reference [26,27], and the primer sequences used are listed in Table S1.

### 2.9. Mycelial Fermentation and UPLC-MS of Ganoderma Triterpenoids

The dikaryon-editing strain G0119-KO-*cyp512a3* and the overexpressing strain L1-OE-*cyp512a3* were respectively inoculated into YMG liquid medium and shaken and cultured at 26 °C and 150 rpm for 10 days, while their corresponding wild-type strains, L1 and G0119, were synchronously cultured under the same conditions. After the culture period, the fermentation broth was transferred to a sterile homogenization cup and subjected to low-speed homogenization for 7 s, repeated twice. Subsequently, 10 mL of the homogenized broth was transferred into a secondary YMG fermentation liquid medium and further shaken and cultured for 5 days, followed by static incubation at 26 °C. The mycelial pellets formed on the surface of the fermentation broth were collected, freeze-dried, and reserved for further use. The freeze-dried mycelial pellets were extracted by adding absolute alcohol at a solid-to-liquid ratio of 1:20 (*w*/*v*), sonicated for 1 h, and then centrifuged at 6000 rpm for 10 min. The supernatant was collected, filtered through a 0.22 μm organic filter membrane, and the filtrate was diluted 100-fold stepwise for subsequent ultra-performance liquid chromatography-mass spectrometry (UPLC-MS) analysis to calculate the changes in the content of individual ganoderic acids in the dry weight of the mycelial pellets. The UPLC-MS detection of Ganoderma triterpenoids was performed according to the method of Yue et al. [28]. The concentrations of the target analytes were determined by means of an Agilent UPLC system (1290 infinity II) coupled with an Agilent triple quadrupole mass spectrometer (6495 Triple Quad(Agilent Technologies, Santa Clara, CA, USA)). The LC-MS system was controlled with Agilent MassHunter Workstation software (version B.08.00). More specifically, optimization of the precursor ions, quantitative ions, and qualitative ions of all reference standards was completed by means of the Agilent MassHunter Optimizer (version B.08.00). The chromatography and retention time determination were performed by Agilent MassHunter Qualitative Analysis (version B.07.00). Quantitative calculations and peak integration were performed with Agilent MassHunter Quantitative Analysis (version B.07.01). Gradient elution was achieved through a binary mobile phase mixture of water (mobile phase A, with 0.01% acetic acid) and ACN (mobile phase B). The elution procedure was as follows: 0–5 min, 55% B; 5–25 min, 55–75% B; 25–30 min, 75–85% B; 30–32 min, 85–100% B. The column temperature was set at 35 °C. Additionally, the sample or mixed standard solution was injected at 4 μL via autosampler, with the flow rate maintained at 400 µL/min. UV detection was performed at 240 nm. The sample recovery rate of this method ranged from 93.3% to 109.7%, indicating good accuracy of the method. The relative standard deviation (RSD) of the recovery rate was less than 11.45%, which evaluated the stability of the method at different spiking levels and demonstrated that the quantitative results were reliable.

### 2.10. Functional Characterization of the Gene cyp512a3 in the Triterpene Synthesis Pathway of G. Lucidum Mycelium

To determine the potential functional position of *cyp512a3* in the triterpene biosynthetic pathway of *G. lucidum*, we first analyzed the 11 reported *cyp450* genes involved in triterpene synthesis in *G. lucidum* [13,16,17,18,19,20,21,22,23]. Using the website https://www.ncbi.nlm.nih.gov/(accessed on 6 June 2025), we retrieved the corresponding gene identifiers in the L1 genome. Subsequently, MEGA-X software and the website https://itol.embl.de/ (accessed on 6 June 2025) were utilized to construct a phylogenetic tree for the 170 *cyp450* genes within the L1 genome. By integrating the reported triterpene synthesis pathway in *G. lucidum* mycelium [13,16,17,18,19,20,21,22,23,29,30] and the clustering relationship of *cyp512a3* with *cyp450* genes of known functions in the phylogenetic tree, we ultimately inferred the potential functional position of *cyp512a3* in the triterpene synthesis pathway of *G. lucidum* mycelium.

## 3. Results and Analysis

### 3.1. Design of sgRNA and Verification of RNP Cleavage Efficiency in Vitro

**Figure 2:**
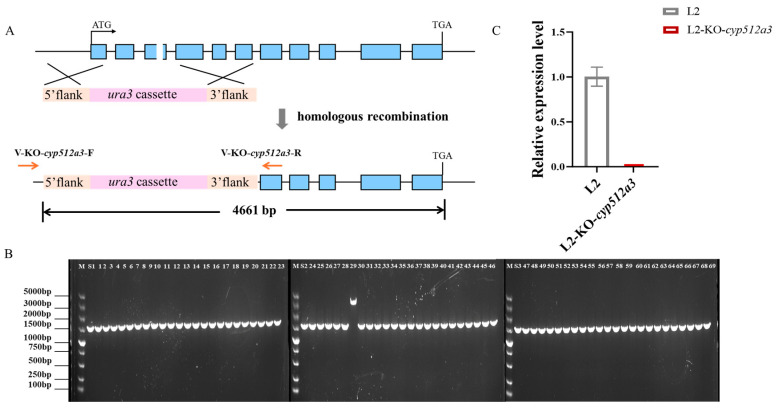
Acquisition of *cyp512a3* editing strain. (**A**) Schematic diagram of homologous recombination of donor DNA for *cyp512a3*. The blue sections represent gene exons, the pink section is the *ura3* expression cassette, and the orange sections are the homologous upper arm and homologous lower arm. (**B**) Gel electrophoresis image for the verification of 69 putative transformants. M represents the 5000 molecular weight marker; S1, S2, and S3 are the control strain L2; 1~69 are the putative transformants. (**C**) Detection of RT-qPCR expression levels of the *cyp512a3* gene in the L2 monokaryotic strain and the L2-KO-*cyp512a3* monokaryotic edited strain.

The full length of the *cyp512a3* gene is 2120 bp, comprising a total of 11 exons (Supplementary Data S1). The selected sgRNA sequence is 5′-GACGAGGATTTATCGGCTCC-3′, and its cleavage site is located 419 bp downstream of the start codon ATG (Figure 2A, Supplementary Data S1). Subsequently, an in vitro transcription template for the sgRNA was constructed (the sequence is shown in Supplementary Data S2), and in vitro transcription was carried out. To verify whether the Cas9 protein guided by this sgRNA could specifically cleave the target sequence of *cyp512a3*, an in vitro cleavage experiment was conducted. Theoretically, the RNP complex composed of the Cas9 protein and sgRNA-*cyp512a3* can cleave the 2120 bp target fragment, generating two products of 419 bp and 1701 bp (Figure 1A). Agarose gel electrophoresis results showed that after the addition of the RNP complex, the 2120 bp fragment was successfully cleaved, producing two bands that matched the expected sizes (approximately 1701 bp and 419 bp; Figure 1B). This indicates that the prepared RNP complex can effectively cleave the target sequence of *cyp512a3*.

### 3.2. Acquisition of G. lucidum L2-KO-cyp512a3 Gene Editing Strain

During the gene editing of *cyp512a3* in strain L2, RNP-*cyp512a3* (220.6 nM) and donor DNA (15 μg) were co-transformed into L2-Δ*ura3* protoplasts (10^7^ in number). Screening was conducted on minimal medium lacking uracil: the RNP complex induced double-strand breaks in the target sequence, while the donor DNA facilitated homologous recombination via its homologous arms, complementing the *ura3* gene near the target site. This enabled the transformants to grow on minimal medium deficient in uridine, thus achieving the screening process (Figure 2A). The sequence of the donor DNA is provided in Supplementary Data S3. A total of 69 putative transformants were screened using minimal medium. After genomic DNA extraction and PCR analysis, only one strain exhibited a band consistent with the expected size (Figure 2B). Sequencing of this transformant (results shown in Supplementary Data S4) revealed that homologous recombination had occurred between the sequences near the target site and the 5′ and 3′ flanking sequences of the donor DNA, successfully achieving knockout of the *cyp512a3* gene and complementation of the *ura3* gene. This edited strain was named L2-KO-*cyp512a3*. Further qRT-PCR results demonstrated that, compared to the wild-type L2, the expression level of the *cyp512a3* gene in L2-KO-*cyp512a3* was extremely low and almost undetectable (Figure 2C).

### 3.3. The Dikaryon-Editing Strain cyp512a3 Was Obtained by Single–Single Hybridization

**Figure 3:**
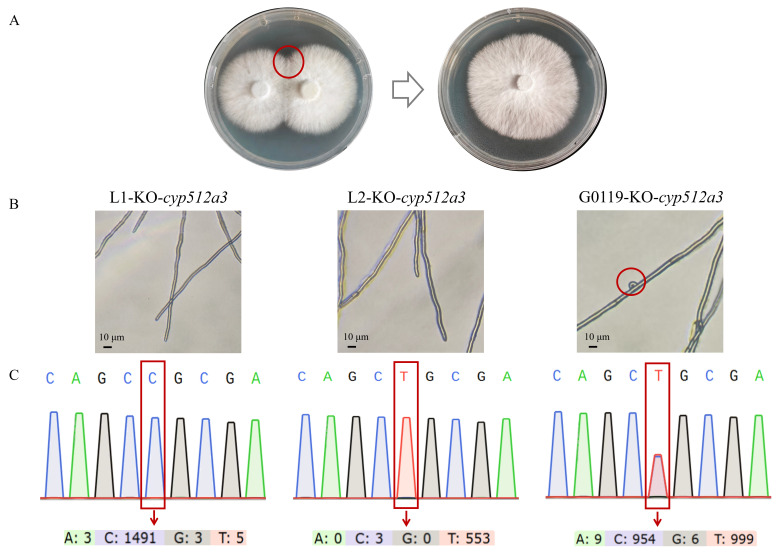
Single–single hybridization of *cyp512a3* monokaryotic editing strain. (**A**) Schematic diagram of the confrontation culture plate of strains L1-KO-*cyp512a3* and L2-KO-*cyp512a3*. (**B**) Mycelial morphology of strains L1-KO-*cyp512a3*, L2-KO-*cyp512a3* and G0119-KO-*cyp512a3* under a microscope. The red circle indicates the presence of clamp connections in the dikaryotic strain G0119-KO-*cyp512a3*. (**C**) DNA sequencing chromatograms of strains L1-KO-*cyp512a3*, L2-KO-*cyp512a3* and G0119-KO-*cyp512a3*.

L1 and L2 are two monokaryotic strains with compatible mating types isolated from G0119. The *cyp512a3* gene was knocked out in L1, and the strain was designated L1-KO-*cyp512a3* (obtained in previous experiments in our laboratory). The *cyp512a3* gene was knocked out in L2, and the strain was designated L2-KO-*cyp512a3* (obtained in this study). The two edited monokaryotic strains L1-KO-*cyp512a3* and L2-KO-*cyp512a3* were co-cultured by confrontation assay. Mycelia were picked from the contact zone and re-cultured to form a new colony (Figure 3A). In the newly formed colonies, the typical structure of dikaryotization in basidiomycetes, known as clamp connection, was observed, whereas this structure was not found in the two monokaryotic parent strains (Figure 3B). Further SNP locus analysis revealed that the new colonies exhibited overlapping peaks at 60 bp after the ATG of the *ura3* gene, while both monokaryotic parent strains showed single peaks at this locus (Figure 3C). The aforementioned results indicate that L1-KO-*cyp512a3* and L2-KO-*cyp512a3* have successfully mated and formed dikaryotic mycelium, with the *cyp512a3* gene knocked out in both nuclei of this dikaryotic strain. This strain was named G0119-KO-*cyp512a3* and was used for subsequent research (results shown in Supplementary Data S5).

### 3.4. Acquisition of G. lucidum L1-OE-cyp512a3 Overexpression Strain

**Figure 4:**
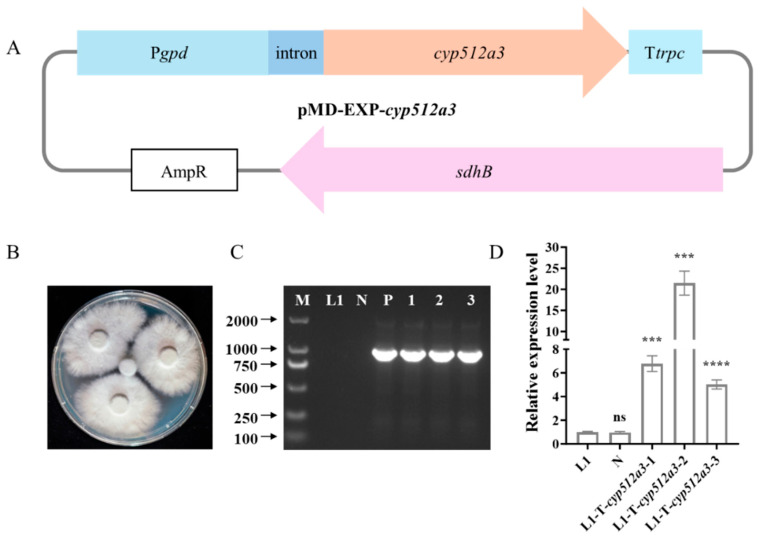
Acquisition of *cyp512a3* overexpressing strains. (**A**) Plasmid map of pMD-EXP-*cyp512a3*. (**B**) Three stable resistant transformants were obtained on PDA medium containing carboxin; the L1 control strain showed no growth in the middle. (**C**) Agarose gel electrophoresis image for verification of resistant transformants. M represents the 2000 DNA molecular weight marker; the L1 monokaryotic strain serves as the control; N is the negative control (the L1 monokaryotic strain transformed with an empty vector not containing the target gene); P is the positive control (the fragment amplified from the plasmid pMD-EXP-*cyp512a3*); and 1, 2, and 3 are the fragments amplified from the three resistant transformants L1-T-*cyp512a3*-1, L1-T-*cyp512a3*-2, and L1-T-*cyp512a3*-3, respectively. (**D**) RT-qPCR detection of strains. The L1 monokaryotic strain serves as the control; N is the L1 monokaryotic strain transformed with an empty vector not containing the target gene; and L1-T-*cyp512a3*-1, L1-T-*cyp512a3*-2, and L1-T-*cyp512a3*-3 are the three resistant transformants. ****, indicates statistical significance (*p* < 0.0001) compared to the L1 strain. ***, indicates statistical significance (*p* < 0.001) compared to the L1 strain. ns, indicates no statistical significance (*p* > 0.05) compared to the L1 strain.

The pMD-EXP-*cyp512a3* plasmid (Figure 4A) was transformed into the *G. lucidum* strain L2. After 20 days of cultivation, a total of 22 resistant transformants were obtained on carboxin-containing selection plates. Ten of these transformants were randomly selected and inoculated onto fresh PDA medium. Following five rounds of subculturing, they were screened again on PDA medium containing carboxin, ultimately yielding three stable resistant transformants (Figure 4B). The integration of the target gene into the transformants was verified through PCR and sequencing. The results showed that all three resistant transformants could amplify the expected bands encompassing the P*gpd* promoter, intron, and a portion of the *cyp512a3* sequence (Figure 4C), whereas no corresponding bands were observed in the negative control. The sequencing results of the resistant transformants are presented in Supplementary Data S6. The three transformants were named L1-T-*cyp512a3*-1, L1-T-*cyp512a3*-2, and L1-T-*cyp512a3*-3, respectively. qRT-PCR analysis revealed that, compared to the wild-type L1 strain, the expression levels of the *cyp512a3* gene in L1-T-*cyp512a3*-1, L1-T-*cyp512a3*-2, and L1-T-*cyp512a3*-3 were increased to 6.8-fold, 21.5-fold, and 5.0-fold, respectively (Figure 4D). The expression of *cyp512a3* in the empty vector control strain showed no significant difference from that in L1, indicating that the expression vector itself did not affect the transcription of the target gene. Based on these findings, the L1-T-*cyp512a3*-2 strain, which exhibited the highest expression level, was selected for subsequent experiments and renamed as L1-OE-*cyp512a3*.

### 3.5. Triterpene Changes Caused by Knockout and Overexpression of Cyp512a3 Gene

**Figure 5:**
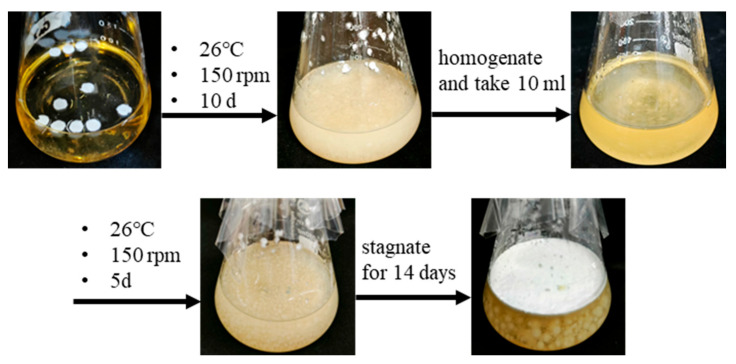
Strain fermentation flow chart.

**Figure 6:**
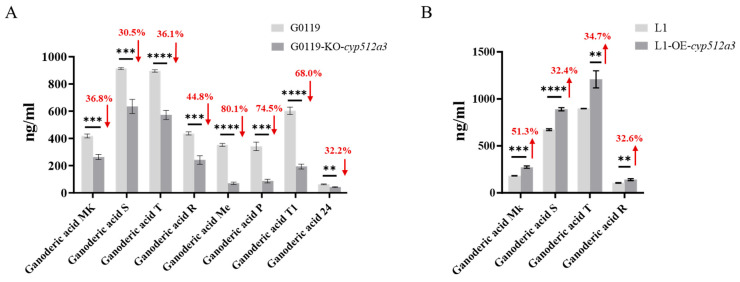
UPLC-MS detection results of fermented mycoderma of G0119-KO-*cyp512a3* and L1-OE-*cyp512a3* strains. (**A**) Changes in ganoderic acid content in the fermented mycoderma of the G0119-KO-*cyp512a3* strain. ****, indicates statistical significance (*p* < 0.0001) compared to the G0119 strain. ***, indicates statistical significance (*p* < 0.001) compared to the G0119 strain. **, indicates statistical significance (*p* < 0.01) compared to the G0119 strain. (**B**) Changes in ganoderic acid content in the fermented mycoderma of the L1-OE-*cyp512a3* strain. ****, indicates statistical significance (*p* < 0.0001) compared to the L1 strain. ***, indicates statistical significance (*p* < 0.001) compared to the L1 strain. **, indicates statistical significance (*p* < 0.01) compared to the L1 strain.

The dikaryotic knockout strain G0119-KO-*cyp512a3*, the overexpression strain L1-OE-*cyp512a3*, along with their wild-type strains G0119 and L1, were cultivated using a two-stage process involving shaking followed by stationary culture, as illustrated in the flowchart in Figure 5. After 14 days of stationary culture, the upper mycelial mat was collected to extract ganoderic triterpenoids. The content of ganoderic triterpenoids was then determined using UPLC-MS. The results showed that, compared to strain L1, the concentrations of various ganoderic acids in the overexpression strain L1-OE-*cyp512a3* significantly increased, with ganoderic acid Mk, ganoderic acid S, ganoderic acid T, and ganoderic acid R increasing by 51.3%, 32.4%, 34.7%, and 32.6%, respectively (Figure 6A). Compared to strain G0119, the concentrations of eight ganoderic acid components in the dikaryotic edited strain G0119-KO-*cyp512a3* decreased to varying degrees: ganoderic acid Me decreased by 80.1%, ganoderic acid P by 74.5%, ganoderic acid T1 by 68.0%, ganoderic acid R by 44.8%, ganoderic acid Mk by 36.8%, ganoderic acid T by 36.1%, ganoderic acid 24 by 32.2%, and ganoderic acid S by 30.5% (Figure 6B). The above results indicate that *cyp512a3* is a key *cyp450* gene influencing the synthesis of triterpenoids in the mycelium of *G. lucidum*. Through calculations, it was found that the contents of ganoderic acid Mk, ganoderic acid S, ganoderic acid T, and ganoderic acid R in the L1-OE-*cyp512a3* strain were 0.548 ± 0.020, 1.780 ± 0.028, 2.416 ± 0.148, and 0.281 ± 0.016 mg/g dry weight of mycelial mat, respectively, which were 1.5, 1.3, 1.3, and 1.3 times those of the L1 control strain, respectively. The contents of ganoderic acid Mk, ganoderic acid S, ganoderic acid T, ganoderic acid R, ganoderic acid Me, ganoderic acid P, ganoderic acid T1, and ganoderic acid 24 in the G0119-KO-*cyp512a3* strain were 0.528 ± 0.031, 1.269 ± 0.085, 1.144 ± 0.055, 0.482 ± 0.050, 0.140 ± 0.014, 0.174 ± 0.022, 0.386 ± 0.030, and 0.085 ± 0.006 mg/g dry weight of mycelial mat, respectively, which were 0.6, 0.7, 0.6, 0.6, 0.2, 0.3, 0.3, and 0.7 times those of the G0119 control strain, respectively (Table 1).

### 3.6. Functional Localization of Gene Cyp512a3 in Triterpenoid Synthesis Pathway of G. lucidum mycelia

**Figure 7:**
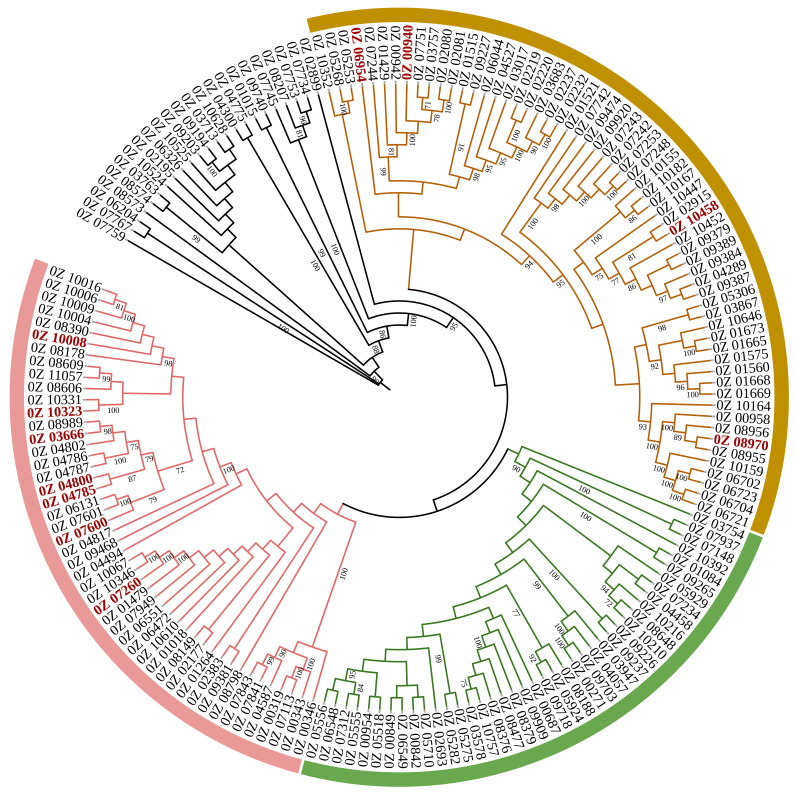
The phylogenetic tree prediction of CYP genes in *G. lucidum* G0119. The genes marked in red represent those that have been reported in Table 2.

The gene IDs corresponding to 11 previously reported *cyp450* genes involved in the synthesis of triterpenoids in *G. lucidum* were retrieved from the L1 genome (Table 2). A phylogenetic tree was constructed using 170 *cyp450* genes from the L1 genome (Figure 7), and the results revealed that *cyp512a3* (*0Z_04785*) had the closest evolutionary relationship with *cyp512a13* (*0Z_04800*), the latter of which is known to hydroxylate the C12 position and oxidize the hydroxyl group at the C15 position of TIIGAs [20]. Additionally, *cyp512a3* was also evolutionarily close to *cyp512w2* (*0Z_03666*) and *cyp512a2* (*0Z_07600*). Among them, *cyp512w2* can catalyze the conversion of TIGAs to TIIGAs, transform HLDOA into GA-Y, and mediate oxidation at the C15 and C11 positions [18,20]; *cyp512a2* can similarly convert TIGAs to TIIGAs and transform HLDOA into GA-Y, but its catalytic activity is lower than that of *cyp512w2* [18,29].

**Figure 8:**
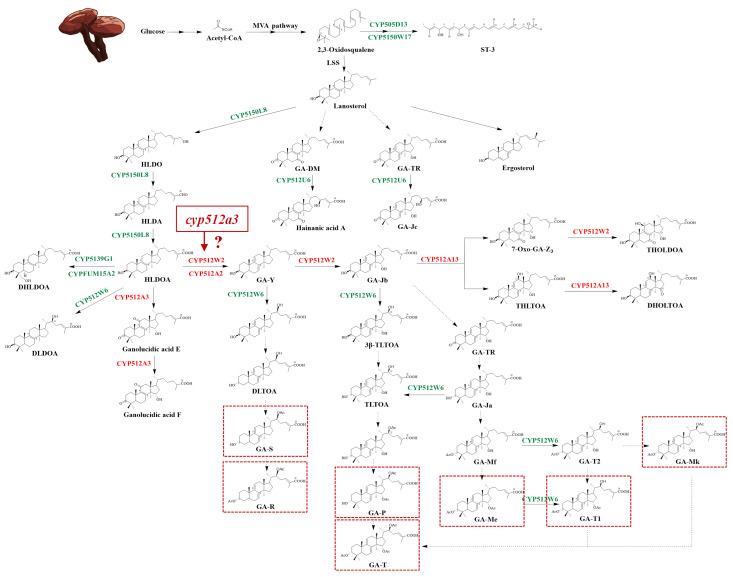
Prediction of triterpenoid biosynthesis pathway of CYP genes in *G. lucidum* G0119.

Based on the known functions of the aforementioned three homologous genes, it is speculated that *cyp512a3* may possess similar partial catalytic activities and thus participate in the regulation of the biosynthesis of triterpenoids in *G. lucidum*. The results of this study show that knocking out *cyp512a3* leads to significant changes in the content of eight ganoderic acids, with the positions of seven of these acids in the known triterpenoid synthesis pathway depicted in Figure 8. Notably, both *cyp512w2* and *cyp512a2* act on the same node downstream of the ganoderic acid HLDOA, and blocking this node may affect the synthesis of the aforementioned seven ganoderic acids. Combining the evolutionary relationships with metabolic phenotypes, it is inferred that *cyp512a3* (*0Z_04785*) may also act on this key node, thereby participating in the synthesis and regulation of eight mycelial triterpenoids in *G. lucidum*, including ganoderic acid Mk.

## 4. Discussion

This study systematically explores the role of the *cyp450* gene *cyp512a3* in the biosynthesis of triterpenes in *G. lucidum* using an endogenous system through gene editing and overexpression techniques for the first time. Although Du et al. [23] confirmed that *cyp512a3* can catalyze the conversion of ganoderic acid HLDOA to ganolucidic acid E and F using a heterologous expression system (*Saccharomyces cerevisiae*), it is worth noting that these products are not abundant triterpenoid compounds found in the mycelium of *G. lucidum*. This actually reflects a common phenomenon in studies of *cyp450* genes related to triterpenes in *G. lucidum* using heterologous systems: due to differences in metabolic backgrounds, the triterpene products generated through heterologous expression often deviate significantly from the endogenous metabolic profile of *G. lucidum*. This, to some extent, limits the translation of research results into industrial applications. In contrast, conducting gene function studies within the endogenous environment of *G. lucidum* has significant advantages: by regulating endogenous genes, it is possible to directly alter the levels of major triterpene compounds in the mycelium. These compounds not only have well-defined biological activities but also align closely with the metabolic regulatory mechanisms required for mycelial fermentation production. Furthermore, this study provides more direct experimental evidence for elucidating the synthesis pathways of key triterpene compounds during the mycelial stage, and these results can directly support the targeted breeding of high-yield strains and the optimization of fermentation processes.

As an important edible and medicinal fungus, the development of *G. lucidum* products primarily relies on the use of its dikaryotic fruiting bodies and the spores they produce [31,32,33]. Therefore, gene editing of dikaryotic strains of *G. lucidum* has significant industrial application value. However, when gene editing is performed directly on dikaryotic strains, it often results only in heterozygous edited strains with a single nucleus being edited [34,35]. This may be due to intrinsic differences between the two nuclei, leading to uneven editing efficiency. Recently, Choi et al. [36] achieved a major breakthrough in *Ganoderma lucidum* gene editing by directly performing CRISPR/Cas9 editing on dikaryotic strains. They obtained 31 transformants, among which only 2 strains showed simultaneous editing in both nuclei. In this study, a stepwise strategy was employed: first, the *cyp512a3* gene was knocked out in two mating-type compatible monokaryotic strains (L1 and L2), and then the dikaryotic edited strains were obtained through single mating. The editing method used in this study features high editing efficiency and low screening cost. The editing efficiency of monokaryotic strains can reach 100% [34], and screening only needs to be performed against the haploid background, resulting in a low workload. In contrast, for dikaryotic editing, the probability of simultaneous targeting of both nuclei is compounded, leading to high screening costs for obtaining effective homozygous strains, which require extensive validation to acquire the target strains. Furthermore, unlike the study by Choi et al. [36], we co-transformed protoplasts with both RNP complexes and donor DNA, enabling the donor DNA to mediate homology-directed repair (HR) of double-strand breaks (DSB). Accordingly, the dikaryotic edited strains of *Ganoderma lucidum* obtained in this study were homozygous, which is more conducive to the functional analysis of specific target genes in subsequent research. In this study, we used *ura3* as the marker gene, and a monokaryotic strain with the *ura3* gene knocked out was used as the starting strain. The functional gene *cyp512a3* was knocked out via the CRISPR/Cas9-mediated cleavage of the target gene, accompanied by the complementation of the *ura3* gene. Strains with successful *ura3* complementation could synthesize uridine, which is essential for growth, and thus grow on medium lacking uridine, whereas strains without successful complementation could not grow. Based on this selectable characteristic of the *ura3* gene, we successfully obtained the *cyp512a3*-knockout strain. This system lays a foundation for the study of other functional genes in the future. In contrast, Choi et al. [36] only knocked out the *pyrG* marker gene, and the application of marker gene-based research to the study of functional genes may still be a long process. In addition, the method employed in this study supports stepwise stacking editing in separate nuclei, where different genes are edited in distinct monokaryotic strains, followed by hybridization to generate a dikaryon. In contrast, multigene co-editing in dikaryotic strains is extremely difficult, making it challenging to achieve precise reconstruction of metabolic pathways. In conclusion, his method offers stable heritable dikaryotic edited materials for the functional gene research of *G. lucidum*. More importantly, this study provides an essential dikaryotic editing tool for exploring important life traits of *G. lucidum*, such as fruiting body development, lignin degradation, and spore powder production. The in-depth analysis of these traits holds even greater biological significance compared to the study of triterpenoid synthesis pathways. Additionally, previously reported gene editing studies on edible fungi such as *Lentinula edodes* and *Flammulina Filiformis* have all been conducted in monokaryotic materials [37,38,39,40]. The editing strategy established in this paper can also serve as a methodological reference for the study of key life traits in other edible fungi like *Lentinula edodes* and *Flammulina Filiformis*.

According to the results in Figure 8, it is speculated that the *cyp512a3* gene may be positioned upstream of lanosterol in the triterpenoid synthesis pathway of *G. lucidum*, located at a critical node in the metabolic network. After knocking out the *cyp512a3* gene, the content of ganoderic acid Me significantly decreased, suggesting that this gene may be involved in key steps of the synthesis pathway of ganoderic acid Me, such as hydroxylation or oxidation reactions at specific positions [18,22]. It is noteworthy that the changes in the ganoderic acid profile caused by the overexpression of the *cyp512a3* gene were more limited than those observed in the knockout treatment. This phenomenon can be explained by the complexity of the metabolic pathway: while overexpressing a single enzyme gene can accelerate specific reaction steps, if the cell cannot simultaneously enhance the supply of precursor substances and the level of energy metabolism, the overall flux of the pathway will still be constrained. In contrast, knocking out a key gene may block the core metabolic pathway, often resulting in more significant metabolic disturbances. This phenomenon of “limited overexpression effects and significant knockout effects” is relatively common in complex secondary metabolic pathways, reflecting the overall coordination and bottleneck effects of the metabolic network. In overexpression strains, excessive expression of the target gene driven by a strong promoter often triggers a series of non-physiological effects, including metabolic disorders caused by protein misfolding and aggregation, aberrant subcellular localization of proteins that further interferes with normal transcriptional regulation, and non-specific binding of overexpressed target proteins to other metabolic enzymes, thereby inhibiting their enzymatic activities. In contrast, the metabolic changes exhibited by gene knockout strains mainly represent physiological adaptations of cells to the loss of gene function, without the aforementioned artificial artifacts derived from overexpression. Consequently, the metabolic phenotypes of overexpression and knockout strains naturally display significant asymmetry.

Furthermore, in our previous work, a CRISPR/Cas9 gene-editing system based on RNP was successfully established for *Ganoderma lucidum*, with further optimization achieved by the addition of Triton X-100. In this established system, DNA double-strand breaks (DSBs) were repaired through the non-homologous end joining (NHEJ) pathway, and the editing efficiency for the *ura3* gene reached over 35 mutants per 10^7^ protoplasts—confirming that the constructed gene-editing system is stable and reliable [24]. In the present study, we introduced donor DNA to facilitate DSB repair via homologous recombination (HR); however, the HR efficiency in *Ganoderma lucidum* remains extremely low. This low efficiency is primarily attributed to the fact that DSB repair in *Ganoderma lucidum* is predominantly dependent on the NHEJ pathway rather than HR. As the initiator of the NHEJ pathway, the Ku70/Ku80 heterodimer can rapidly bind to DSB ends and initiate NHEJ-mediated repair, thereby suppressing the HR pathway [41,42]. However, knockout of Ku70/Ku80 leads to growth defects [43]. Additionally, the expression levels of Rad51 and Rad52, which are key genes involved in the HR pathway, are relatively low in *Ganoderma lucidum*. Even when the NHEJ pathway is inhibited, the activation of HR remains inadequate. In future research, we will attempt to enhance HR efficiency by synchronizing the cell cycle, optimizing the selection of Cas proteins and the design of single-guide RNAs (sgRNAs), and refining the construction of donor DNA.

## 5. Conclusions

In this study, the monokaryotic *Ganoderma lucidum* strain L2 with the *ura3* gene knocked out was used as the recipient strain. Using an RNP-mediated CRISPR/Cas9 genome editing system and donor DNA, the functional gene *cyp512a3* was successfully knocked out. Subsequently, a homozygous dikaryotic knockout strain of *cyp512a3* was obtained via monokaryon–monokaryon hybridization. Compared with the wild-type strain, the contents of several ganoderic acids were significantly decreased in the edited strain. Based on phylogenetic relationships and metabolic phenotypes, the potential catalytic site of *cyp512a3* was predicted. This study provides a practical strategy for obtaining dikaryotic *Ganoderma lucidum* strains with both nuclei edited and offers important support for exploring the biosynthetic pathway of ganoderic acids and gene function.

## Figures and Tables

**Table 1:** Comparison of dry weight content of individual ganoderic acids in mycelial mat (mg/g dry weight).

	G0119 (mg/g)	G0119-KO-*cyp512a3* (mg/g)	L1 (mg/g)	L1-OE-*cyp512a3* (mg/g)
GA-MK	0.835 ± 0.023	0.528 ± 0.031	0.362 ± 0.003	0.548 ± 0.020
GA-S	1.826 ± 0.011	1.269 ± 0.085	1.345 ± 0.017	1.780 ± 0.028
GA-T	1.790 ± 0.015	1.144 ± 0.055	1.794 ± 0.003	2.416 ± 0.148
GA-R	0.874 ± 0.019	0.482 ± 0.050	0.212 ± 0.004	0.281 ± 0.016
GA-Me	0.705 ± 0.017	0.140 ± 0.014	—	—
GA-P	0.683 ± 0.052	0.174 ± 0.022	—	—
GA-T1	1.205 ± 0.044	0.386 ± 0.030	—	—
GA-24	0.125 ± 0.005	0.085 ± 0.006	—	—

**Table 2:** Eleven reported *cyp450* genes involved in triterpene synthesis of *G. lucidum*.

Gene Name	ID	Functions Reported (In Yeast Heterologous Expression System)	Reference
*cyp512u6*	*0Z_10008*	Catalytic hydroxylation of *G. lucidum* acid DM and TR at C-23 position.	[16]
*cyp505d13*	*0Z_07260*	Catalytic oxidation of squalene to produce squalene-type triterpenoids (STs).	[17]
*cyp5150w17*	*0Z_08970*	Catalytic oxidation of squalene to produce 2,3; 22,23-squalene dioxide (ST-3).	[17]
*cyp512w2*	*0Z_03666*	The oxidation reaction of GA-HLDOA was catalyzed to produce GA-Y and GA-Jb, which laid a foundation for the industrial production of type II ganoderic acid.	[18]
*cypfum15a2*	*0Z_06954*	The C-28 methyl group of GA-HLDOA was oxidized to produce a new ganoderic acid derivative, 3,28-dihydroxylanosta-8,24-diene-26-oic acid (DHLDOA).	[18]
*cyp512a2*	*0Z_07600*	The oxidation reaction of GA-HLDOA was catalyzed to produce GA-Y, which laid a foundation for the industrial production of type II ganoderic acid.	[18]
*cyp5150l8*	*0Z_10458*	The three-step oxidation reaction of lanosterol at the C-26 position was catalyzed to produce GA-HLDOA.	[13,19]
*cyp512a13*	*0Z_04800*	It is a key enzyme for the formation of ketene structure and cooperates with cyp512w2 to achieve de novo synthesis of new type I GAs (such as THOLDOA).	[20]
*cyp5139g1*	*0Z_00940*	The C-28 methyl group of GA-HLDOA was oxidized to produce a new ganoderic acid derivative (DHLDOA).	[21]
*cyp512w6*	*0Z_10323*	Catalytic hydroxylation at the C22 position in the biosynthesis pathway of type II ganoderic acid (TIIGAs).	[22]
*cyp512a3*	*0Z_04785*	It catalyzes the oxidation of GA-HLDOA to ganolucidic acid E and ganolucidic acid F.	[23]

## Data Availability

The original contributions presented in this study are included in the article/supplementary material. Further inquiries can be directed to the corresponding authors.

## Supplementary Materials

The following supporting information can be downloaded at: https://www.mdpi.com/article/10.3390/jof12030183/s1, Supplementary Data S1: Sequences amplified from the L2 genome using primers L2-*cyp512a3*-F/R; Supplementary Data S2: The sgRNA transcription cassettes (T7 promoter-Spacer-sgRNA scaffold) of *cyp512a3*; Supplementary Data S3: Donor DNA sequences of *cyp512a3*; Supplementary Data S4: Sequences of the mutant edited through the *G. lucidum* CRISPR system, amplified in L2-Δ*cyp512a3* using primers V-KO-*cyp512a3*-F/R; Supplementary Data S5: Sequences amplified in L1-KO-*cyp512a3* and L2-KO-*cyp512a3* using primers *ura3*-SNP-F/R; Supplementary Data S6: Sequences amplified from the L1 genome using primers V-OE- *cyp512a3*-F/R. Table S1: Primer sequences.
