# Supplementary material for: Development of a CRISPR/Cas9 Genome Editing System in Dikaryotic Ganoderma lucidum for Targeting Key CYP450 Gene Involved in Triterpenoid Synthesis
Source: J Fungi (Basel). 2026 Mar 4;12(3):183. doi: 10.3390/jof12030183 (PMC13027530; doi:10.3390/jof12030183)
Supplement: Supplementary file 1 [file jof-12-00183-s001.zip › jof-4122728-supplementary.pdf]

Supplementary Materials

Supplementary Data S1: Sequences amplified from the L2 genome using primers L2-*cyp512a3*-F/R. The exons were marked with red letters. The PAM sequence and sgRNA-guiding sequence were highlighted in yellow and green, respectively. The red triangle indicated the cleavage site.

ATGACAGTGGAGGATCCTCAAGCTCTCATTCTGGCCGGCGTCGCCATCCTCGCTGTCCT
ATACGTCGTCCGATGGCAGACAGACCCCGTAAGTGGGTCCTCACAAAGCGCTATGGGG
TCAATGATAAACTAATTATCCGAACAGCTGAGGTCTATCCCCACAGTTGGTGGGCCGTC
CGCACCAGGACTATCGATGCTGTCAGCGCTCAATTACCTGCGTAATGGGAAGAAGGTG
ATGACTGAGGGCTACCGGAAGGTCTGTCTATCTTCTCCACCGGCAGCTCCGCAGGGAC
TCTGCAGGAGCTCACAAATCCTGTTTAATAGTACCACGGAGGAGCCTTCAAGGTCGCA
CAGCTCGACAAATGGGTAGTCGTGGTTTCCGGGCGCAAGTTGGTCGACGAACTCTGGC
GGCGACCGGACGAGGATTTATCGGCTCCTGCGGCTGTTCAGGACGTATGAATACGCAT
GCAGCCGCACCTCTTGTCCTACATTGACTTGGTCTCCTTCGTAGATCATCCAGATGAGA
TATACTCTCGGACACGAAGCATTTGATGACCCGTATGAAACCGACATCATCAAGGAGA
AGCTTACGCGTTCGCTTCCGGTCATTTTTCCTGACGTCGTCGAGGAGATGAGGCTTGCC
GTGTCGGACTACATCCCCACAAAAGGAGACGGTAAGCAGTCTTTCAACCAGACCCTCT
TTTGACGGGCTCTCAACTGAATTCACAAAGAGTGGACCCCTGTCAATGGTATGGACAC
GGCGGTGAATATTGTCGCAAGAACCAGTAGCCGCGCCTTCGTGGGTCTCCCACTTTGT
AGGTCCTCATTTTCACTTCATCAGCATACCGACCAGCTCATGCGGAAACGCTTCAGGCC
GCAACGAAGAATTCTTGGCGTTGATTCTTCGTTTCACTATGGATGTGTTGAAGGATCGG
TTCTTTCTCAACACTTTCCCGTACTTTTTGAAGCCGTGAGTCCAGTATTGGAAAGTAAT
GACAGGATTTCTCACGTTCTCTGGACAGTTACTTCGGGCACGCCTTCAGTAGAGCGAA
GAGGACTATCTATCAAGGACTGACATTCCTCCAACCGTTGATCACCGAAAGAAGGATG
AATATGCGAGAGCTTGGGGACGACTGGTCTGACAAGCCGGTAAGAGTCCCGTCGAGG
GTCTGCGACAATGTGACTAAAGAGTGCCTGTTGCAGGTTGATATGCTTCAGTGGGTCG
TGGAAGCGGCAATGGCCAGGAAGAGCGATGATTATAGGATTGCGGAACGCATGTTCCT
CGTTAACTTCGCAGCTCTCCACACAACCTCCACCGTGCGCCCAATCTTCCCTGTCCTGC
CAAGCGGTACTAATACCCTAGTTCAACCCGTATAGACCCTTGCGCACGCGCTCTATGAT
CTCGCTGCCATGCCCGAGCTCATTCCAGAACTGCGGGAAGAGGTCGATTCCGCCATCG
CGTCGGACGGTTGGTCCAAAGCGGCTGTAGGCAAAATGTGGAAGCTCGATAGCGTGT
GCAGAGAGGCGTTGCGTTACCATGGAATGAGCTTCAGTGTGTATCATATTATCTGTTAC
ACGAGCAGGCTCAGCGGTTCCCTGACTGACGGCCTACACCCCCCCCCTCCCACCAGT
CGGTCTTTTCCGCAAGGCAATGAAGGACGTCACGCTGAGCGACGGGACATTCATCCCG
AAAGGCACCACCGTAGTCGCCGCAGCAGGCCCCACGCACCACGAGGCGTCGATCTAC
CCCAACCCGGAGGTGCTCGACCCCTTCCGCTTCGCAAAGTTGGGTGCAAGTGGCGGA
GAAGGCGGTTCCGTGAAGCTCCAAACCGTTTCCACGTCCATTGATTTCCTGCCCTTCG
GCCATGGCAAGCACGCTTGGTATGTCCTCTCTCCGCCACGCCCTGCTCGTGCTAAATCA
AGCGTTCTGACTGCCGTACAGCCCGGGACGGTGGTTCGCGGCAAACGAGGTGAAGAT
GATCTTAGCGCACATCGTGCTCAACTACGACCTGAAGCTCGGTGGAGACGGCAAACGG
CCTGGGGACACGCTCTTCGGTACCACCATACTTCCGCCCCGTGGGCAGGTATATTTCAG
GAAACGCAAGGGGGTGTCCGAGTGA

Supplementary Data S2: The sgRNA transcription cassettes (T7 promoter-Spacer-sgRNA scaffold) of *cyp512a3*.

TTCTAATACGACTCACTATAGACGAGGATTTATCGGCTCCGTTTTAGAGCTAGAAATAG
CAAGTTAAAATAAGGCTAGTCCGTTATCAACTTGAAAAAGTGGCACCGAGTCGGTGCT
TTT

Supplementary Data S3: Donor DNA sequences of *cyp512a3*. The sequence of 5' flank, *ura3* cassette and 3' flank was marked with green, red and blue letters, respectively.

ATTTAGCAGCTGCTGTTCCCCGTTCCTCATTCTCGCTCCATGGCATGATCAACCTCGCTT
TCTAGGCTTTACATCGGGGCATGCACGGTCATCCAGGGGCATGCGTGGCAACTTAAATC
GACATGGGCTTCCAGTAACCCGGCGAAAACAGACATACCTGTGCGGTCTGAGCAAATG
AAGCAAGTGCCCTACCAAACCATCCTATATCTCGGAGCGCGGTCACCTGGATCAGTAG
ATGTGAGTAAGGACCATTACACACGCAACCTTCCATACGCCCCAGAGTCGTCCGAGTT
GGCAGCAGGCATGCGTCGCCATAAGTATCCTCTATCACCTTCCGCCTATCTTGTTACTTC
CCTCCTCTCTCAGCCAACTGGACCGTTGGACTCGTGCGCCATCTCTCTGATGACAGTG
GAGGATCCTCAAGCTCTCATTCTGGCCGGCGTCGCCATCCTCGCTGTCCTATACGTCGT
CCGATGGCAGACAGACCCCGTAAGTGGGTCCTCACAACTCCACTCCCTCCAACTATTT
TCGCCTGTCGGCCTGCCCAGCCGCTGTCACTCACGAATGGCGGTTCTGGTCCCTCAAG
ATACAGGTCCTTGACACGGGCAATAAGGGCTTCTGACAAGAAGGGTTTGAGTGATTCA
GTGAAGCGTTCGGGCCATGGCTCGTCCTCTTGTGGAGTGTCCCCGTCAACTTTTCGTTC
ATCCGTTGCGGGTGGCTGCGCGGCGTCCGGTGCGGGGTCGGCGACAACAGCGTCGCT
AACTTGCTCTGCAGTGGTGGCAGAAGTTTCGGGCTCGTTGTTCTCACCGACGGCTTGA
TCAGCTGGCTTAGACGTAGTGGAGACTCCTGCAGGCGTCACATCCGCCCCGTTCTTCG
ATGAGGATGGCATAGCGAGCGTCTTCAAATGGATGACCTGACTGTCTTGGTCGACCTC
GAATACCAAGAAGTCCGTGAACCTGGAAGGCCATATGAACTAAATAAACGAACGCGA
GTATGACCTTACCACGCACCTCTGCTTGATGATGCCCTCGATTTTGGGCACATCAAAAC
CAATGTACTCAGATATACCAACGTCCGTTTCCATGATTTGCTGTATAGAGCCATCTGGAC
CATGCACTGGGGGTTTTGTGTTGAGCAGCGCATGACTTGGCGGAAGAAGAGTCTCCGG
TTCGTCGTTCTTCACGTCAACGTCCATATCGTCCTGTTCCTGCTCTTCGACGTCGGCGAT
GCTGACAACCGTGACGGTGCTGTCCGTGCTCATAGGCATGACTCCGTCCCCGTCCTCTA
CTTTTGCTCGCTTGGCATTCGGTTCATCCGCTAGATCTGGCTCGCGAGGGCGAGTTTTT
GGCGAGGACGACATTCGGTGTCAGTGAAGCGGAGGGGAGTAGAGACGGTCACTGGA
GAGATGGGAGAGCCTCGTTCGAGGCAAAACTGGAAAACAGCGCGCTTATCTAATCTCA
CAAGTCACCCAGCAACCACCCGTCGGCCACAAAACAAAATTGAAAGAATGGTGGCCG
TGGCCAAGCAAACATACGCGCAGAGGGCCACCAGACATCCCAACCCAGCTGCGAAAG
CTCTCCTCGAGACGATCGAGCGCAAGCGCACAAATCTGTCTGTTAGCGTCGATGTGAC
GAAACGGGAGGACTTCTTCAGGATTGTGGACATCGTGGGTCCATACGTCTGCCTAGTA
AAGGTAGGTGTACTATGTCCAAATGGTTGATATGCACTTGGGTGAATATAATCCAAAGA
CCCACATAGACATCATCGAGGACTTTGATCCATCCGCGATCGAACGCCTCAAGGCACTC
AGCGAGAAGCATGACTTCCTCATCTTTGAAGACAGAAAATTTGCCGACATTGGTAGGA
TGTAGTCCCTTTGCGCCCGCTACATACGATGTTGACAAGAATATAGGAAACACGGTGGC
ACTTCAATATTCTGCAGGCGTGCACAAAATCGCTAGCTGGTCGCACATCACGAACGCA
CACCCTGTCCCAGGACCGTCCATCATCTCCGGCCTCAAGGCTGTCGGTCTGCCCCTTG

GCAGGGGGCTTCTGCTCCTCGCCGAGATGAGCACCAAGGGCAGCCTCGCAACCGGCT
CATACACGGAAGAGGCCGTCCGTATGGCTCGCGCCAACCGCGACTTCGTGATCGGCTT
CATTGCCCAGCAACGCATGGACAGCGTCGGTCTGCGGGAGGGCGAGTCCTCGCCGGA
CGAAGACTTCCTTATCCTTACTCCAGGAGTCGGACTGGATACTAGGGGAGATAGCATGG
GGCAGCAATACCGAACGCCAAGGGAGGTGGTCATCGAGTCCAATTGCGATGTCATCAT
AGTTGGTCGGGGTGTTTACGGGAACGATAACGGCACGAATGCGGAGGCAGTCCGCGC
ACAGGCGGAGAGGTACCGCGCAGAAGGCTGGAAGGCATACCAGGAAAGGGTTGGGA
TCTCGGATTAGGGCTAGCATTGTGCAGAGACGGGTATAGTAGCACAGTGTATATGTAAA
CAAAACATCCTACCGCTACAATCGCAATACATATACACATACTTATCGTTAAATCAGGCA
GTCGTTCCTTCCACTTCCGCCTTCCTTCTCTCCTCTTCCTCTTTGGCTTCGCGCTCTTCT
TCCTCTACAACTTCGATGTCCAGTGGGAAGGTCAAAATTGCAGCGATGCCCGTTAGTT
GGTTCAACTCTGCATGAAATTGACCATGTCAGTGGTACGGTAAGCATCAGGATGGGAA
AACTCTTACGCTGGCCAGACTCGTGCATACTAGAGAAGATCAAGACCTCCCCGCCTTT
CTGTTGGACGTCTTCCACTATGCGCACGTACTTTTTGCGCACCGTAGCGTTGCTTGACC
TGGGATTATGATCAAGACTACGTTCATCGGAAGGAGTCAGCTGGGCTAACCTGAACAA
CTCGTCCGAGATCATAAGTGTGCCGATGGCGCCTCGATCTGAAGCAAGAGCGACATGA
TCGGGACCATACCAGGCTCGCATCTCGTCCGAGGCTAGCATCTTGAAGAACCTGTGAC
ATTGACTTGGTCTCCTTCGTAGATCATCCAGATGAGATATACTCTCGGACACGAAGCATT
TGATGACCCGTATGAAACCGACATCATCAAGGAGAAGCTTACGCGTTCGCTTCCGGTC
ATTTTTCCTGACGTCGTCGAGGAGATGAGGCTTGCCGTGTCGGACTACATCCCCACAA
AAGGAGACGGTAAGCAGTCTTTCAACCAGACCCTCTTTTGACGGGCTCTCAACTGAAT
TCACAAAGAGTGGACCCCTGTCAATGGTATGGACACGGCGGTGAATATTGTCGCAAGA
ACCAGTAGCCGCGCCTTCGTGGGTCTCCCACTTTGTAGGTCCTCATTTTCACTTCATCA
GCATACCGACCAGCTCATGCGGAAACGCTTCAGGCCGCAACGAAGAATTCTTGGCGTT
GATTCTTCGTTTCACTATGGATGTGTTGAAGGATCGGTTCTTTCTCAACACTTTCCCGTA
CTTTTTGAAGCCGTGAGTCCAGTATTGGA

Supplementary Data S4: Sequences of the mutant edited through the *G. lucidum* CRISPR system, amplified in L2-Δ*cyp512a3* using primers V-KO-*cyp512a3*-F/R. The sequence of 5' flank, *ura3* cassette and 3’ flank was marked with green, red and blue letters, respectively.

CTTACGCCTCTTCATCCCCATTTATATTGCACCATGGAATTTAGCAGCTGCTGTTCCCCG
TTCCTCATTCTCGCTCCATGGCATGATCAACCTCGCTTTCTAGGCTTTACATCGGGGCAT
GCACGGTCATCCAGGGGCATGCGTGGCAACTTAAATCGACATGGGCTTCCAGTAACCC
GGCGAAAACAGACATACCTGTGCGGTCTGAGCAAATGAAGCAAGTGCCCTACCAAAC
CATCCTATATCTCGGAGCGCGGTCACCTGGATCAGTAGATGTGAGTAAGGACCATTACA
CACGCAACCTTCCATACGCCCCAGAGTCGTCCGAGTTGGCAGCAGGCATGCGTCGCCA
TAAGTATCCTCTATCACCTTCCGCCTATCTTGTTACTTCCCTCCTCTCTCAGCCAACTGG
ACCGTTGGACTCGTGCGCCATCTCTCTGATGACAGTGGAGGATCCTCAAGCTCTCATTC
TGGCCGGCGTCGCCATCCTCGCTGTCCTATACGTCGTCCGATGGCAGACAGACCCCGTA
AGTGGGTCCTCACAACTCCACTCCCTCCAACTATTTTCGCCTGTCGGCCTGCCCAGCCG
CTGTCACTCACGAATGGCGGTTCTGGTCCCTCAAGATACAGGTCCTTGACACGGGCAA
TAAGGGCTTCTGACAAGAAGGGTTTGAGTGATTCAGTGAAGCGTTCGGGCCATGGCTC
GTCCTCTTGTGGAGTGTCCCCGTCAACTTTTCGTTCATCCGTTGCGGGTGGCTGCGCGG
CGTCCGGTGCGGGGTCGGCGACAACAGCGTCGCTAACTTGCTCTGCAGTGGTGGCAG
AAGTTTCGGGCTCGTTGTTCTCACCGACGGCTTGATCAGCTGGCTTAGACGTAGTGGA
GACTCCTGCAGGCGTCACATCCGCCCCGTTCTTCGATGAGGATGGCATAGCGAGCGTC
TTCAAATGGATGACCTGACTGTCTTGGTCGACCTCGAATACCAAGAAGTCCGTGAACC
TGGAAGGCCATATGAACTAAATAAACGAACGCGAGTATGACCTTACCACGCACCTCTG
CTTGATGATGCCCTCGATTTTGGGCACATCAAAACCAATGTACTCAGATATACCAACGT
CCGTTTCCATGATTTGCTGTATAGAGCCATCTGGACCATGCACTGGGGGTTTTGTGTTG
AGCAGCGCATGACTTGGCGGAAGAAGAGTCTCCGGTTCGTCGTTCTTCACGTCAACGT
CCATATCGTCCTGTTCCTGCTCTTCGACGTCGGCGATGCTGACAACCGTGACGGTGCTG
TCCGTGCTCATAGGCATGACTCCGTCCCCGTCCTCTACTTTTGCTCGCTTGGCATTCGGT
TCATCCGCTAGATCTGGCTCGCGAGGGCGAGTTTTTGGCGAGGACGACATTCGGTGTC
AGTGAAGCGGAGGGGAGTAGAGACGGTCACTGGAGAGATGGGAGAGCCTCGTTCGA
GGCAAAACTGGAAAACAGCGCGCTTATCTAATCTCACAAGTCACCCAGCAACCACCCG
TCGGCCACAAAACAAAATTGAAAGAATGGTGGCCGTGGCCAAGCAAACATACGCGCA
GAGGGCCACCAGACATCCCAACCCAGCTGCGAAAGCTCTCCTCGAGACGATCGAGCG
CAAGCGCACAAATCTGTCTGTTAGCGTCGATGTGACGAAACGGGAGGACTTCTTCAGG
ATTGTGGACATCGTGGGTCCATACGTCTGCCTAGTAAAGGTAGGTGTACTATGTCCAAA
TGGTTGATATGCACTTGGGTGAATATAATCCAAAGACCCACATAGACATCATCGAGGAC

TTTGATCCATCCGCGATCGAACGCCTCAAGGCACTCAGCGAGAAGCATGACTTCCTCA
TCTTTGAAGACAGAAAATTTGCCGACATTGGTAGGATGTAGTCCCTTTGCGCCCGCTAC
ATACGATGTTGACAAGAATATAGGAAACACGGTGGCACTTCAATATTCTGCAGGCGTGC
ACAAAATCGCTAGCTGGTCGCACATCACGAACGCACACCCTGTCCCAGGACCGTCCAT
CATCTCCGGCCTCAAGGCTGTCGGTCTGCCCCTTGGCAGGGGGCTTCTGCTCCTCGCC
GAGATGAGCACCAAGGGCAGCCTCGCAACCGGCTCATACACGGAAGAGGCCGTCCGT
ATGGCTCGCGCCAACCGCGACTTCGTGATCGGCTTCATTGCCCAGCAACGCATGGACA
GCGTCGGTCTGCGGGAGGGCGAGTCCTCGCCGGACGAAGACTTCCTTATCCTTACTCC
AGGAGTCGGACTGGATACTAGGGGAGATAGCATGGGGCAGCAATACCGAACGCCAAG
GGAGGTGGTCATCGAGTCCAATTGCGATGTCATCATAGTTGGTCGGGGTGTTTACGGGA
ACGATAACGGCACGAATGCGGAGGCAGTCCGCGCACAGGCGGAGAGGTACCGCGCAG
AAGGCTGGAAGGCATACCAGGAAAGGGTTGGGATCTCGGATTAGGGCTAGCATTGTGC
AGAGACGGGTATAGTAGCACAGTGTATATGTAAACAAAACATCCTACCGCTACAATCGC
AATACATATACACATACTTATCGTTAAATCAGGCAGTCGTTCCTTCCACTTCCGCCTTCC
TTCTCTCCTCTTCCTCTTTGGCTTCGCGCTCTTCTTCCTCTACAACTTCGATGTCCAGTG
GGAAGGTCAAAATTGCAGCGATGCCCGTTAGTTGGTTCAACTCTGCATGAAATTGACC
ATGTCAGTGGTACGGTAAGCATCAGGATGGGAAAACTCTTACGCTGGCCAGACTCGTG
CATACTAGAGAAGATCAAGACCTCCCCGCCTTTCTGTTGGACGTCTTCCACTATGCGCA
CGTACTTTTTGCGCACCGTAGCGTTGCTTGACCTGGGATTATGATCAAGACTACGTTCA
TCGGAAGGAGTCAGCTGGGCTAACCTGAACAACTCGTCCGAGATCATAAGTGTGCCGA
TGGCGCCTCGATCTGAAGCAAGAGCGACATGATCGGGACCATACCAGGCTCGCATCTC
GTCCGAGGCTAGCATCTTGAAGAACCTGTGACATTGACTTGGTCTCCTTCGTAGATCAT
CCAGATGAGATATACTCTCGGACACGAAGCATTTGATGACCCGTATGAAACCGACATCA
TCAAGGAGAAGCTTACGCGTTCGCTTCCGGTCATTTTTCCTGACGTCGTCGAGGAGAT
GAGGCTTGCCGTGTCGGACTACATCCCCACAAAAGGAGACGGTAAGCAGTCTTTCAAC
CAGACCCTCTTTTGACGGGCTCTCAACTGAATTCACAAAGAGTGGACCCCTGTCAATG
GTATGGACACGGCGGTGAATATTGTCGCAAGAACCAGTAGCCGCGCCTTCGTGGGTCT
CCCACTTTGTAGGTCCTCATTTTCACTTCATCAGCATACCGACCAGCTCATGCGGAAAC
GCTTCAGGCCGCAACGAAGAATTCTTGGCGTTGATTCTTCGTTTCACTATGGATGTGTT
GAAGGATCGGTTCTTTCTCAACACTTTCCCGTACTTTTTGAAGCCGTGAGTCCAGTATT
GGAAAGTAATGACAGGATTTCTCACGTTCTCTGGACAGTTACTTCGG

Supplementary Data S5: Sequences amplified in L1-KO-*cyp512a3* and L2-KO-*cyp512a3* using primers *ura3*-SNP-F/R. The SNP site was marked with red.

＞Sequences amplified from the L1 genome using primers *ura3*-SNP-F/ R

TTTGGCGAGGACGACATTCGGTGTCAGTGAAGCGGAGGGGAGTAGAGACGGTCACTG
GAGAGATGGGAGAGCCTCGTTCGAGGCAAAACTGGAAAACAGCGCGCTTATCTAATCT
CACAAGTCACCCAGCAACCACCCGTCGGCCACAAAACAAAATTGAAAGAATGGTGGC
CGTGGCCAAGCAAACATACGCGCAGAGGGCCACCAGACATCCCAACCCAGCCGCGAA
AGCTCTCCTCGAGACGATCGAGCGCAAGCGCACAAATCTGTCTGTTAGCGTCGATGTG
ACGAAACGGGAGGACTTCTTCAGGATTGTGGACATCGTGGGTCCATACGTCTGCCTAG
TAAAGGTAGGTGTACTATGTCCAAATGGTTGATATGCACTTGGGTGAATATAATCCAAA
GACCCACATAGACATCATCG

＞Sequences amplified from the L2 genome using primers *ura3*-SNP-F/R

TTTGGCGAGGACGACATTCGGTGTCAGTGAAGCGGAGGGGAGTAGAGACGGTCACTG
GAGAGATGGGAGAGCCTCGTTCGAGGCAAAACTGGAAAACAGCGCGCTTATCTAATCT
CACAAGTCACCCAGCAACCACCCGTCGGCCACAAAACAAAATTGAAAGAATGGTGGC
CGTGGCCAAGCAAACATACGCGCAGAGGGCCACCAGACATCCCAACCCAGCTGCGAA
AGCTCTCCTCGAGACGATCGAGCGCAAGCGCACAAATCTGTCTGTTAGCGTCGATGTG
ACGAAACGGGAGGACTTCTTCAGGATTGTGGACATCGTGGGTCCATACGTCTGCCTAG
TAAAGGTAGGTGTACTATGTCCAAATGGTTGATATGCACTTGGGTGAATATAATCCAAA
GACCCACATAGACATCATCG

Supplementary Data S6: Sequences amplified from the L1 genome using primers V-OE-*cyp512a3*-F/R. The partial promoter sequence, whole intron sequence and partial *cyp512a3* gene sequence were highlighted in green, yellow and red, respectively.

GCAGCTCAGCACAAGTTTCTGTGGTGCTGTTGCAGGCACTGAGGAACGTGAGCAGGC
TTTAGAGGGAGCTAGGCTGGGCGACGTTGGTCTGGGTATGCGAGGAAGACATCGGAG
AGATGGCAAGGGCGAGGGACGGGCCGAGTCTAGGCTGAGGCTGAAGAGTCAGACGC
AACTAGCGCTCATTGGCGGGAGACCGCCCCAGTCCGGCAAGCGCCGAGTGACGCAGG
TGGTGACGCCCGCCAGTCGCCCAGTCCGTAGTAGTGTGCCCGATTGGATCTGCGATAA
CATCGGTCGAGGCGTATAAATGCTCTCTCTGCCCTCTCTCCTTGTCCAGCCTCATCTCCT
TACTCACTCTTCATCCCCCTCTCAACATGCCCGTGAGTCCTGCATCCCCATCGTGCACC
GTATTCACCTCATCGTTTGGCCCCCTTCTCACAGGTCAAGGTCGCTCTAGAGATCTAGC
TAGCTAGATGACAGTGGAGGATCCTCAAGCTCTCATTCTGGCCGGCGTCGCCATCCTCG
CTGTCCTATACGTCGTCCGATGGCAGACAGACCCCGTAAGTGGGTCCTCACAAAGCGC
TATGGGGTCAATGATAAACTAATTATCCGAACAGCTGAGGTCTATCCCCACAGTTGGTG
GGCCGTCCGCACCAGGACTATCGATGCTGTCAGCGCTCAATTACCTGCGTAATGGGAA
GAAGGTGATGACTGAGGGCTACCGGAAGGTCTGTCTATCTTCTCCACCGGCAGCTCCG
CAGGGACTCTGCAGGAGCTCACAAATCCTGTTTAATAGTACCACGGAGGAGCCTTCAA
GGTCGCACAGCTCGACAAATGGGTAGTCGTGGTTTCCGGGCGCAAGTTGGTCGACGA
ACTCTGG

Table S1. Primer sequences

Primer	Sequence (5'→3')
L2-*cyp512a3*-F	ATGACAGTGGAGGATCCTCAAG
L2-*cyp512a3*-R	TCACTCGGACACCCCCTT
cyp512a3-UP-F	ttcggatcttccagagatatcATTTAGCAGCTGCTGTTCCCCGT
cyp512a3-UP-R	GGCTAGCATCTTGAAGAACCTGTGTTGTGAGGACCCACTTACG
cyp512a3-URA3-F	CGTAAGTGGGTCCTCACAACACAGGTTCTTCAAGATGCTAGCC
cyp512a3-URA3-R	CTACGAAGGAGACCAAGTCAATGTCTCCACTCCCTCCAACTATTTTCG
cyp512a3-DOWN-F	CGAAAATAGTTGGAGGGAGTGGAGACATTGACTTGGTCTCCTTCGTAG
cyp512a3-DOWN-R	caactgccgttcgacgatatcTCCAATACTGGACTCACGGCTTC
V-KO-*cyp512a3*-F	CTTACGCCTCTTCATCCCCATT
V-KO-*cyp512a3*-R	CCGAAGTAACTGTCCAGAGAAC
ura3-SNP-F	TTTGGCGAGGACGACATTCG
ura3-SNP-R	CGATGATGTCTATGTGGGTC
P*gpd-intron*-F	aattcgagctcggtaccccgTCCAAGCCGCTCTCATGGCAT
P*gpd-intron*-R	ACCTTGACCTGTGAGAAGGG
OE-*cyp512a3*-F	TCACAGGTCAAGGTCGCTCTAGAGATCTAGCTAGCTAGATGGCAGTGG AGGATCCT
OE-*cyp512a3*-R	CGGTCGGCATCTACTTCCCCCCGGGGGGATCACTCGGGCACCCCTTTG CGTTTC
T*trpc-sdhB*-F	AGTAGATGCCGACCGGGATC
T*trpc-sdhB*-R	ggtcgacgatggactagtccTGCTCTATGTCTTGCCTTGTCT
V-OE-*cyp512a3*-F	GCAGCTCAGCACAAGTTTCTGT
V-OE-*cyp512a3*-R	CCAGAGTTCGTCGACCAACTTG
RT-*RPL4*-F	GTCAACAAGGGCGTTCTCTT
RT-*RPL4*-R	AACAGCGTCTTGAGGAAGGT
RT-*cyp512a3*-F	CCTATACGTCGTCCGATGGC
RT-*cyp512a3*-R	TACTTCCGGTAGCCCTCAGT
